# Revisiting the Obesity–Anaemia Paradox: Inflammation and Iron Homeostasis in the BMI–Haemoglobin Relationship

**DOI:** 10.1002/edm2.70110

**Published:** 2025-09-18

**Authors:** Ali Hemade, Pascale Salameh

**Affiliations:** ^1^ Division of Hematology and Oncology, Department of Internal Medicine American University of Beirut Medical Center, Naef K. Bassile Cancer Institute Beirut Lebanon; ^2^ Faculty of Pharmacy Lebanese University Hadat Lebanon; ^3^ Gilbert and Rose‐Marie Chagoury School of Medicine Lebanese American University Beirut Lebanon; ^4^ Department of Primary Care and Population Health University of Nicosia Medical School Nicosia Cyprus; ^5^ Institut National de Santé Publique d'Épidémiologie Clinique et de Toxicologie‐Liban (INSPECT‐LB) Beirut Lebanon

**Keywords:** anaemia, causal mediation, haemoglobin, inflammation, NHANES, nonlinear models, obesity

## Abstract

**Background:**

Obesity and anaemia are global epidemics with complex, overlapping pathophysiology. While excess adiposity is known to induce chronic inflammation that disrupts iron homeostasis, multiple population studies paradoxically report higher haemoglobin levels and lower anaemia prevalence among obese individuals. The nonlinear and potentially suppressive role of inflammation in this relationship remains understudied.

**Methods:**

We analysed adults aged 18–64 from the 2015–2023 National Health and Nutrition Examination Survey (NHANES). Haemoglobin was modelled as a function of body‐mass index (BMI) using survey‐weighted linear regression with restricted cubic splines. Interactions with log‐transformed CRP were assessed, and ferritin was corrected for inflammation using BRINDA regression‐residual methods. Causal mediation analysis decomposed the total effect of BMI on haemoglobin into indirect (mediated by CRP) and direct effects. Secondary models examined anaemia (Hb < 13.0 g/dL in men, < 12.0 g/dL in women) using logistic regression.

**Results:**

Haemoglobin increased steeply across lower BMI ranges but plateaued above 30 kg/m^2^ (p‐nonlinearity < 0.001). The haemoglobin–BMI curve flattened significantly at higher CRP levels, with strong evidence of interaction (p‐interaction < 0.001). Mediation analysis showed that CRP significantly suppressed the BMI–haemoglobin relationship (ACME = −0.044 g/dL, *p* < 0.001; ADE = 0.216 g/dL, *p* < 0.001). In contrast, BRINDA‐adjusted ferritin mediated < 2% of the association. Logistic models showed that anaemia risk declined sharply with increasing BMI but rose consistently with CRP. Anaemia mediation analysis revealed suppression as well (ACME > 0; ADE < 0), precluding interpretation of proportion mediated.

**Conclusions:**

BMI is positively associated with haemoglobin in a non‐linear, CRP‐dependent fashion. Inflammation significantly suppresses the haematologic benefit of excess adiposity, while inflammation‐adjusted ferritin plays a minimal mediating role. These findings underscore the importance of modelling non‐linearity and correcting iron biomarkers for inflammation when studying obesity‐related anaemia.

## Graphical abstract

Using nationally representative NHANES 2015–2023 data, we found that higher body mass index is associated with lower haemoglobin and increased anaemia risk. This relationship is partly mediated by inflammation, highlighting the role of hepcidin and iron restriction in obesity‐related functional iron deficiency.

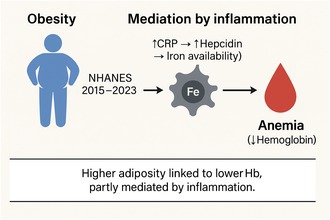

## 1 Introduction

Anaemia remains one of the most pervasive nutritional disorders worldwide. The World Health Organization (WHO) estimates that 30% of women of reproductive age and almost 40% of preschool children are anaemic, with only modest progress toward the 2025 global nutrition target of a 50% reduction in anaemia in women [1]. In parallel, obesity has reached pandemic proportions; more than 42% of U.S. adults are now classified as obese, and global prevalence continues to rise across every region [2, 3]. These twin burdens—frequently co‐existing within the same populations—create complex interactions between excess adiposity, iron metabolism, and red‐cell indices.

A paradoxical pattern has emerged in epidemiologic studies: despite obesity's association with chronic inflammation and disordered iron homeostasis, higher body‐mass index (BMI) often correlates with higher haemoglobin levels and lower anaemia prevalence [4, 5]. Large cross‐sectional surveys in Spain and the United States, for example, report that overweight and obese adults have greater haemoglobin concentrations but also elevated ferritin and hepcidin, biomarkers classically linked to functional iron deficiency [6, 7]. Several explanations have been proposed. First, chronic low‐grade inflammation in obesity stimulates hepatic hepcidin, blocking iron absorption and mobilisation [8, 9, 10]. Second, obesity is accompanied by plasma‐volume expansion that can dilute circulating iron markers while paradoxically increasing total haemoglobin mass [11, 12]. Finally, adiposity is positively correlated with erythropoietin production and erythropoiesis, potentially offsetting hepcidin‐mediated iron restriction [13, 14].

Prior work has important limitations. Most studies rely on linear models of BMI, overlook non‐linear inflection points, and seldom adjust ferritin for inflammation despite guidance from the Biomarkers Reflecting Inflammation and Nutritional Determinants of Anaemia (BRINDA) project [15, 16]. Few have examined whether systemic inflammation, proxied by C‐reactive protein (CRP), modifies the BMI–haemoglobin association or quantifies CRP's mediating role. To address these gaps, we used nationally representative NHANES data (2015–2023) to model the BMI–haemoglobin relation with restricted cubic splines, evaluate effect modification by log‐transformed CRP, and perform mediation analysis after BRINDA adjustment of ferritin. We hypothesised that the BMI–haemoglobin curve would rise and plateau, higher CRP would attenuate haemoglobin gains at upper BMI levels, and CRP would act as a suppressor of the direct positive effect of BMI on haemoglobin.

## 2 Methods

### 2.1 Study Design and Population

We conducted a cross‐sectional study using data from the National Health and Nutrition Examination Survey (NHANES) spanning the 2015 to 2023 cycles. Participants aged ≥ 18 years were eligible if they had valid body mass index (BMI) and haemoglobin (Hb) measurements. Pregnant individuals were excluded. Blood draws were performed at Mobile Examination Centres throughout the day; fasting was not universal for haemoglobin and CRP.

### 2.2 Exposure and Outcomes

BMI was modelled continuously (standardised *z*‐score) and categorically: underweight (< 18.5 kg/m^2^), normal weight (18.5–24.9), overweight (25.0–29.9), obese I (30.0–34.9), obese II (35.0–39.9), and obese III (≥ 40) (WHO/NIH criteria [17]). Haemoglobin concentration (g/dL) was the primary continuous outcome. Anaemia was defined per WHO criteria [18]: Hb < 13.0 g/dL for males and < 12.0 g/dL for females.

### 2.3 Covariates

Covariates included age, sex, race/ethnicity, C‐reactive protein (CRP), and ferritin. Survey cycle‐specific weights were harmonised by dividing the two‐year MEC weights (WTMEC2YR) by four to account for the combined survey cycles.

C‐reactive protein (CRP) values were right‐skewed and therefore log‐transformed as log(CRP + 0.1) to reduce the influence of extreme values and stabilise variance in regression models.

Ferritin, as an acute‐phase reactant, was adjusted for inflammation using the BRINDA (Biomarkers Reflecting Inflammation and Nutritional Determinants of Anaemia) regression correction method [15, 16]. Specifically, residuals from a linear model regressing ferritin on log‐transformed CRP were used as inflammation‐adjusted ferritin estimates (‘BRINDA ferritin’) in downstream analyses.

We did not include clinical conditions such as type 2 diabetes, hepatic disease, or chronic kidney disease in the primary models to avoid over‐adjustment of potential mediators on the adiposity → inflammation → iron axis.

### 2.4 Statistical Analysis

All analyses incorporated the complex survey design of NHANES using the R survey package. We generated weighted means (with SE) for continuous variables and weighted proportions for categorical variables, tabulated by BMI categories (underweight, normal, overweight, obese I/II/III).

#### 2.4.1 Regression Models for Haemoglobin

We fitted survey‐weighted linear regression models using svyglm() to examine the association between BMI and haemoglobin concentration. Haemoglobin was regressed on restricted cubic splines of BMI (ns(BMI, df = 4) with knots at 17.2, 26.4, 37.6 kg/m^2^), adjusting for log(CRP), BRINDA‐adjusted ferritin, age, sex, and race/ethnicity. This model allowed a flexible, non‐linear BMI–haemoglobin relationship and was selected as the primary specification based on improved fit (AIC) and residual diagnostics compared to the linear BMI model. To assess effect modification by inflammation, we extended the spline model by including interactions between each BMI spline basis term and log(CRP). Although our primary focus was on haemoglobin, we ran analogous logistic regression models for anaemia status (binary) with BMI as a spline term to illustrate non‐linear anaemia risk across BMI.

We also conducted mediation analysis to quantify the extent to which CRP mediates the BMI–haemoglobin association. The mediation framework used continuous mediator and outcome models. The mediator model regressed log(CRP) on restricted cubic splines of BMI and covariates; the outcome model regressed haemoglobin on BMI (linear term or spline basis), log(CRP), and covariates. The mediate() function (R mediation package) with 1000 bootstrap replications estimated the average causal mediation effect (ACME), average direct effect (ADE), and total effect. Given the continuous nature of both mediator and outcome, splines could be incorporated safely. We reported that ACME was negative (indicating suppression), ADE positive, and total effect positive (Table 5), and we described this as a suppression effect (‘the indirect path via CRP partially suppresses the positive BMI–haemoglobin association’), omitting any ‘proportion mediated’ when paths were inconsistent.

#### 2.4.2 Model Fit and Assumptions

For each linear model, residuals were examined for homoscedasticity and normality (via survey‐weighted residual plots). Variance inflation factors were checked to rule out multicollinearity among spline terms, CRP, and ferritin. Survey design diagnostics confirmed no highly influential PSUs and realistic design effects. Knot locations for BMI splines (17.2, 26.4, 37.6) were explicitly reported to ensure reproducibility.

All statistical tests were two‐sided, with *p* < 0.05 considered significant. Analyses were performed in R (version 4.4.2) using the survey, splines, and mediation packages. Figures illustrating marginal predicted values and interactions included 95% confidence ribbons and ‘rug’ plots of data density.

## 3 Results

### 3.1 Descriptive Statistics

The analytic sample included 27,048 adults. The weighted anaemia prevalence overall was 10.80% (Table 1a). By BMI category, anaemia prevalence was highest in underweight (30.00%) and lowest in overweight (7.48%) adults (Table 1b). Weighted means for haemoglobin, BMI, CRP, and ferritin are summarised in Table 1c.

**TABLE 1a:** Weighted anaemia prevalence (overall).

Anaemia status	Prevalence (% ± SE)
No	89.20 ± 0.38
Yes	10.80 ± 0.38

**TABLE 1b:** Weighted anaemia prevalence by BMI category.

BMI category	Anaemia no (% ± SE)	Anaemia yes (% ± SE)
Underweight	69.99 ± 1.11	30.00 ± 1.11
Normal	89.80 ± 0.49	10.20 ± 0.49
Overweight	92.52 ± 0.40	7.48 ± 0.40
Obese I	92.29 ± 0.55	7.71 ± 0.55
Obese II	92.00 ± 0.72	8.00 ± 0.72
Obese III	87.88 ± 0.97	12.12 ± 0.97

**TABLE 1c:** Weighted means of biomarkers.

Variable	Mean	SE
Haemoglobin (g/dL)	13.97	0.024
BMI (kg/m^2^)	27.97	0.11
CRP (mg/L)	3.36	0.080
Ferritin (μg/L)	122.86	1.44

### 3.2 Spline Regression

**FIGURE 1:**
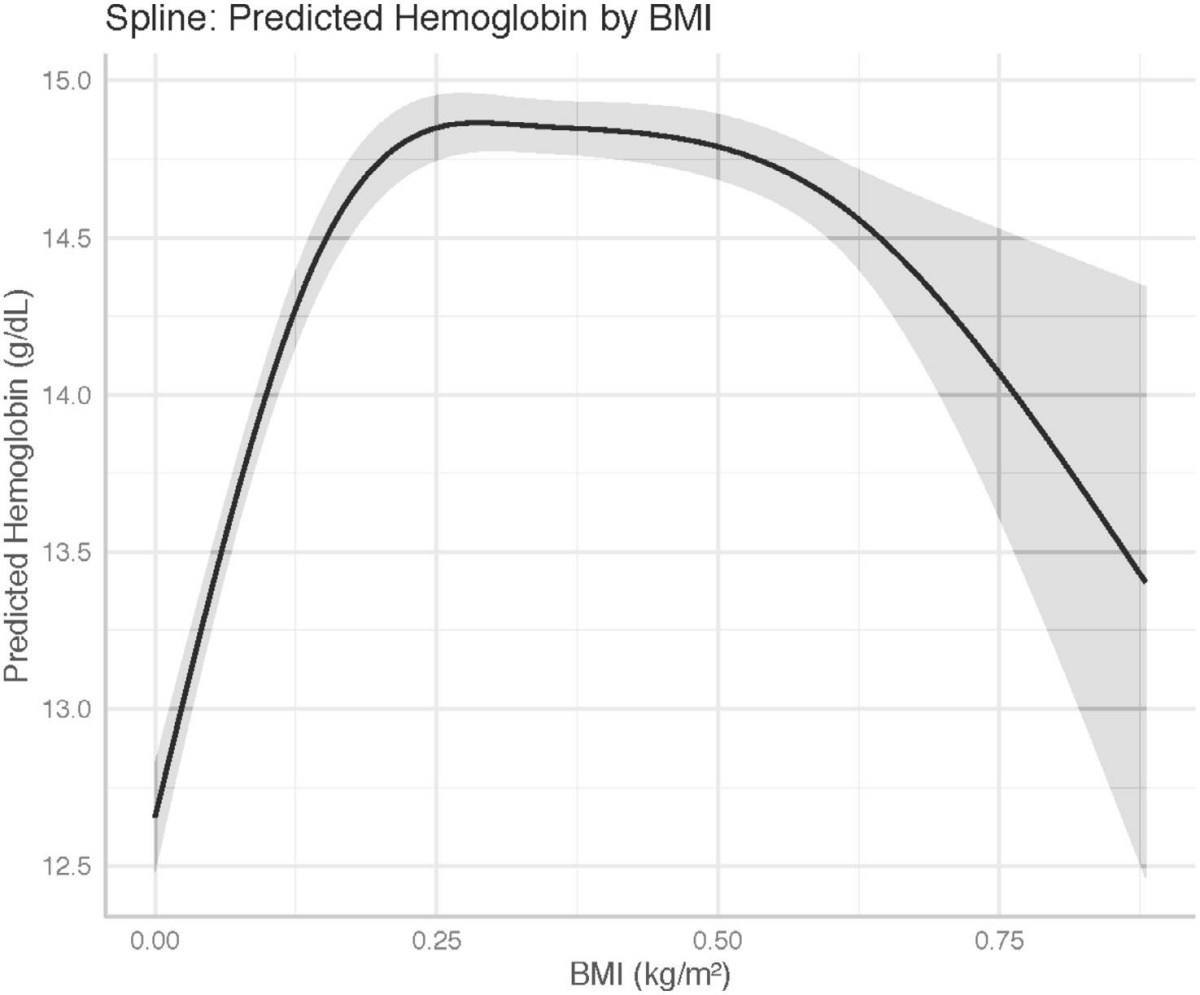
Predicted Haemoglobin Concentration by Body Mass Index (BMI). Spline‐based predictions (solid line) from the survey‐weighted linear model (Model 2a) show the non‐linear association between BMI (kg/m^2^) and haemoglobin (g/dL), adjusting for log‐transformed CRP, BRINDA‐adjusted ferritin, age, sex, and race/ethnicity. Restricted cubic splines with knots at the 10th, 50th, and 90th BMI percentiles (17.2, 26.4, and 37.6 kg/m^2^) were used. The shaded area represents the 95% confidence interval (CI) for predicted haemoglobin at each BMI value, illustrating a steep rise in haemoglobin from BMI 18 to 35 kg/m^2^ and a plateau thereafter.

In fully adjusted spline‐based linear regression models (Table 2), haemoglobin concentration displayed a non‐linear relationship with BMI. Haemoglobin increased sharply across the BMI range from 18 to approximately 35 kg/m^2^, after which the association plateaued. Three of the four spline terms were statistically significant (*p* < 0.001), supporting a curved relationship rather than a constant linear effect. The spline‐based marginal effects plot confirmed this saturating pattern, with predicted haemoglobin rising from ~13.5 g/dL to ~15.0 g/dL, then levelling off beyond BMI 40 kg/m^2^ (Figure 1).

**TABLE 2:** Spline model predicting haemoglobin from BMI.

Variable	Estimate	Std. error	*p*
Intercept	12.66	0.080	< 2e−16***
ns(BMI, df = 4)1	2.17	0.068	< 2e−16***
ns(BMI, df = 4)2	1.58	0.116	< 2e−16***
ns(BMI, df = 4)3	3.14	0.285	2.15e−14***
ns(BMI, df = 4)4	−0.53	0.495	0.291

*Note:* *** indicate statistical significance levels *p* < 0.001.

**FIGURE 2:**
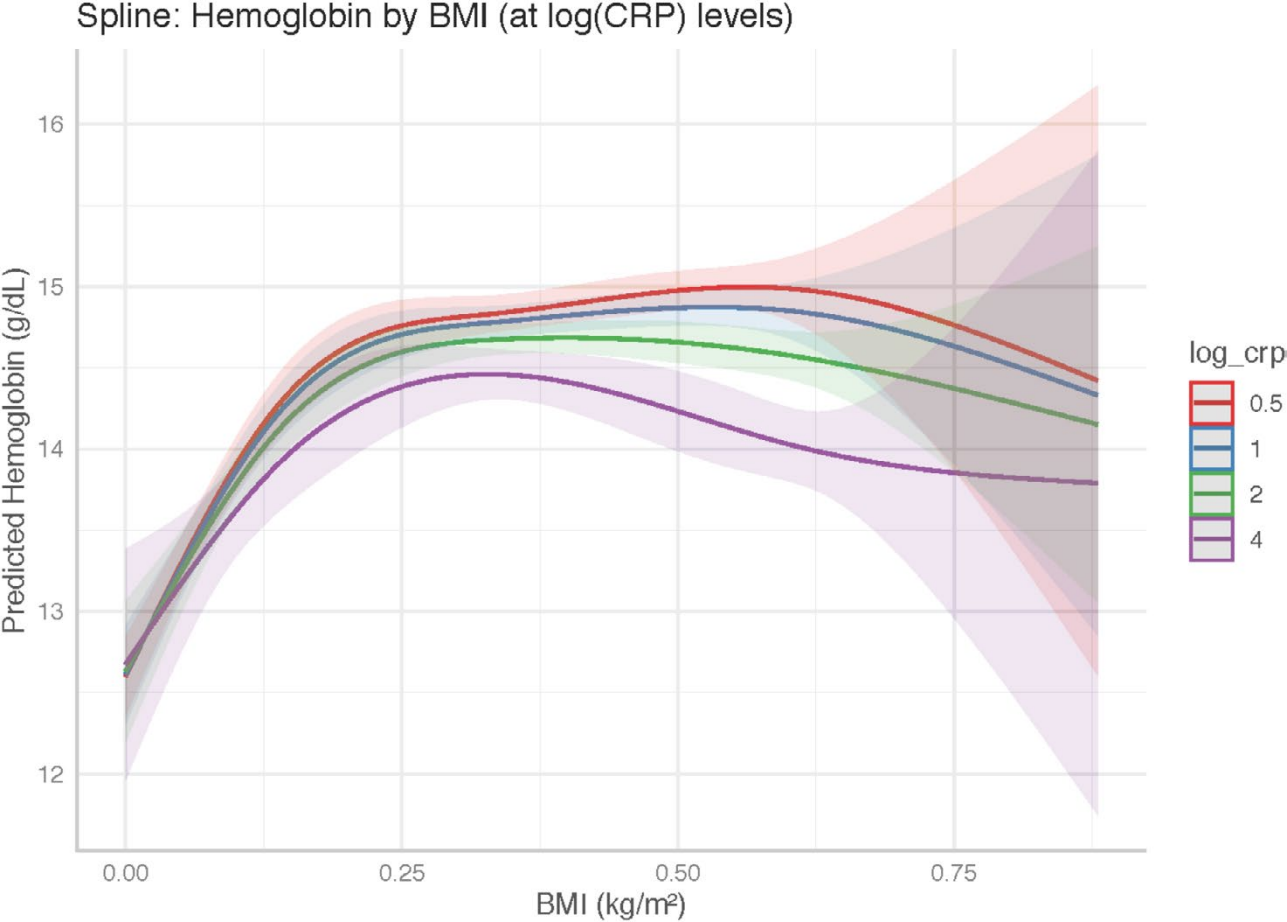
Haemoglobin by BMI at Different log(CRP) Levels. Predicted haemoglobin (g/dL) curves from the spline interaction model (Model 4a) plotted across BMI (kg/m^2^) for four fixed log‐CRP values (0.5, 1, 2, and 4 mg/L). Shaded areas show 95% confidence intervals. Higher inflammation (higher log‐CRP) visibly flattens and eventually reverses haemoglobin gains at higher BMI.

In the spline‐based interaction model including CRP (Table 3), haemoglobin remained non‐linearly associated with BMI. One spline × CRP interaction term was statistically significant (*p* = 0.0002), indicating that the magnitude of BMI's effect on haemoglobin was modified by inflammation. As visualised in Figure 2, individuals with higher CRP levels (log(CRP) > 2) exhibited markedly attenuated haemoglobin gains across the upper BMI range, compared to those with lower inflammation.

**TABLE 3:** Spline interaction model: haemoglobin ~BMI × log(CRP).

Variable	Estimate	Std. error	*p*
Intercept	12.58	0.089	< 2e−16***
ns(BMI, df = 4)1	2.22	0.080	< 2e−16***
ns(BMI, df = 4)2	2.16	0.172	1.71e−15***
ns(BMI, df = 4)3	4.10	0.599	3.00e−08***
ns(BMI, df = 4)4	0.65	1.167	0.579
log(CRP)	0.021	0.072	0.768
ns(BMI, df = 4)1 × log(CRP)	−0.060	0.076	0.436
ns(BMI, df = 4)2 × log(CRP)	−0.341	0.084	0.0002***
ns(BMI, df = 4)3 × log(CRP)	−0.413	0.294	0.167
ns(BMI, df = 4)4 × log(CRP)	−0.102	0.490	0.836

*Note:* *** indicate statistical significance levels *p* < 0.001.

### 3.3 Marginal Effects of BMI and CRP on Haemoglobin and Anaemia

**FIGURE 3:**
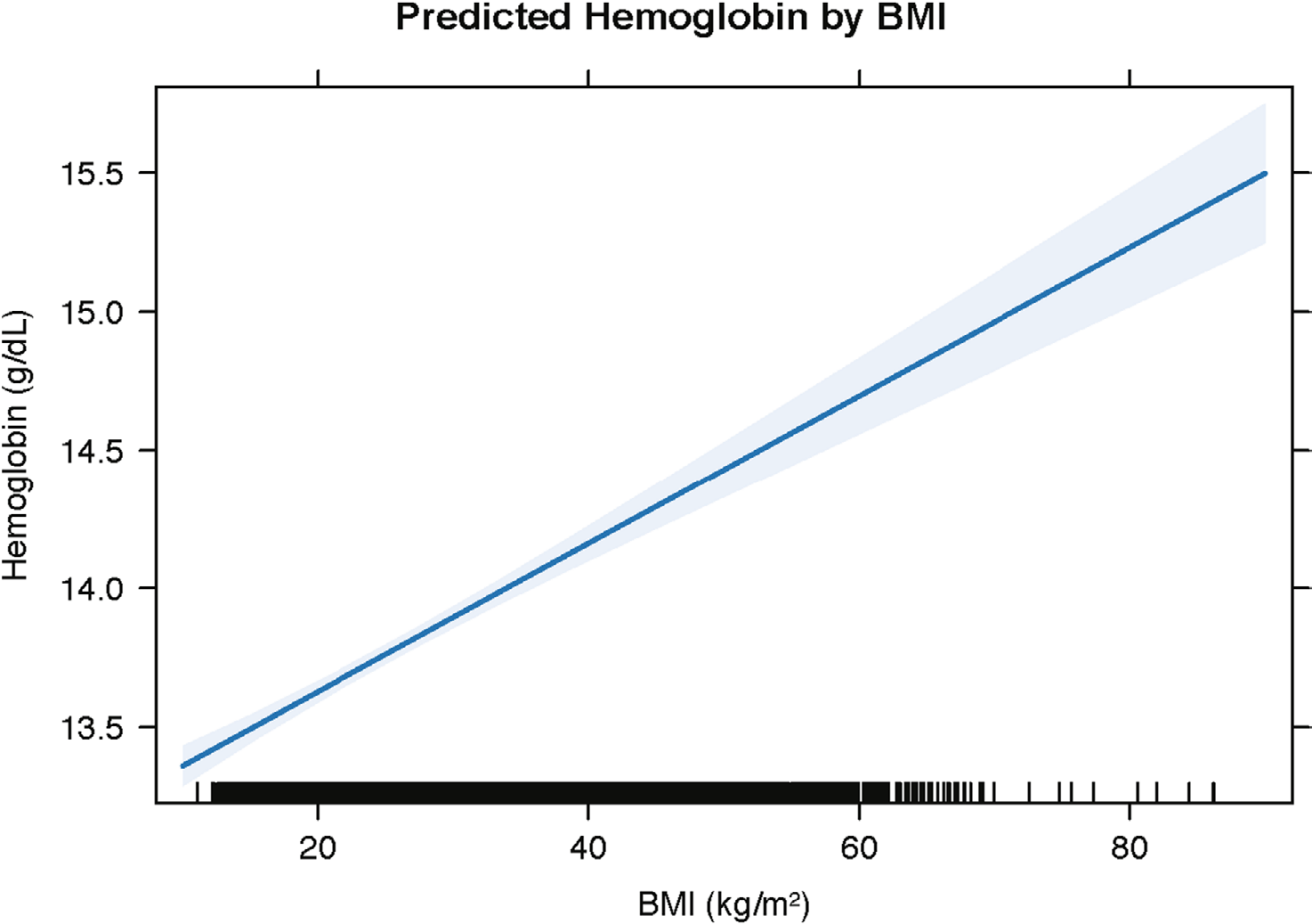
Predicted haemoglobin by BMI (linear model). Marginal predictions (solid line) from the survey‐weighted linear regression of haemoglobin (g/dL) on BMI (kg/m^2^), adjusting for log‐CRP, BRINDA‐adjusted ferritin, age, sex, and race/ethnicity. Shaded area denotes the 95% confidence interval. Tick marks along the *x*‐axis (‘rug’) represent individual BMI values, illustrating data density across the BMI range.

Figure 3 shows the marginal predictions of haemoglobin concentration across the observed range of BMI values, based on the fully adjusted linear model. A clear positive association was observed: haemoglobin levels increased with BMI, with a predicted mean of approximately 13.6 g/dL at a BMI of 20 kg/m^2^ and rising to over 15.0 g/dL at BMI levels exceeding 60 kg/m^2^.

**FIGURE 4:**
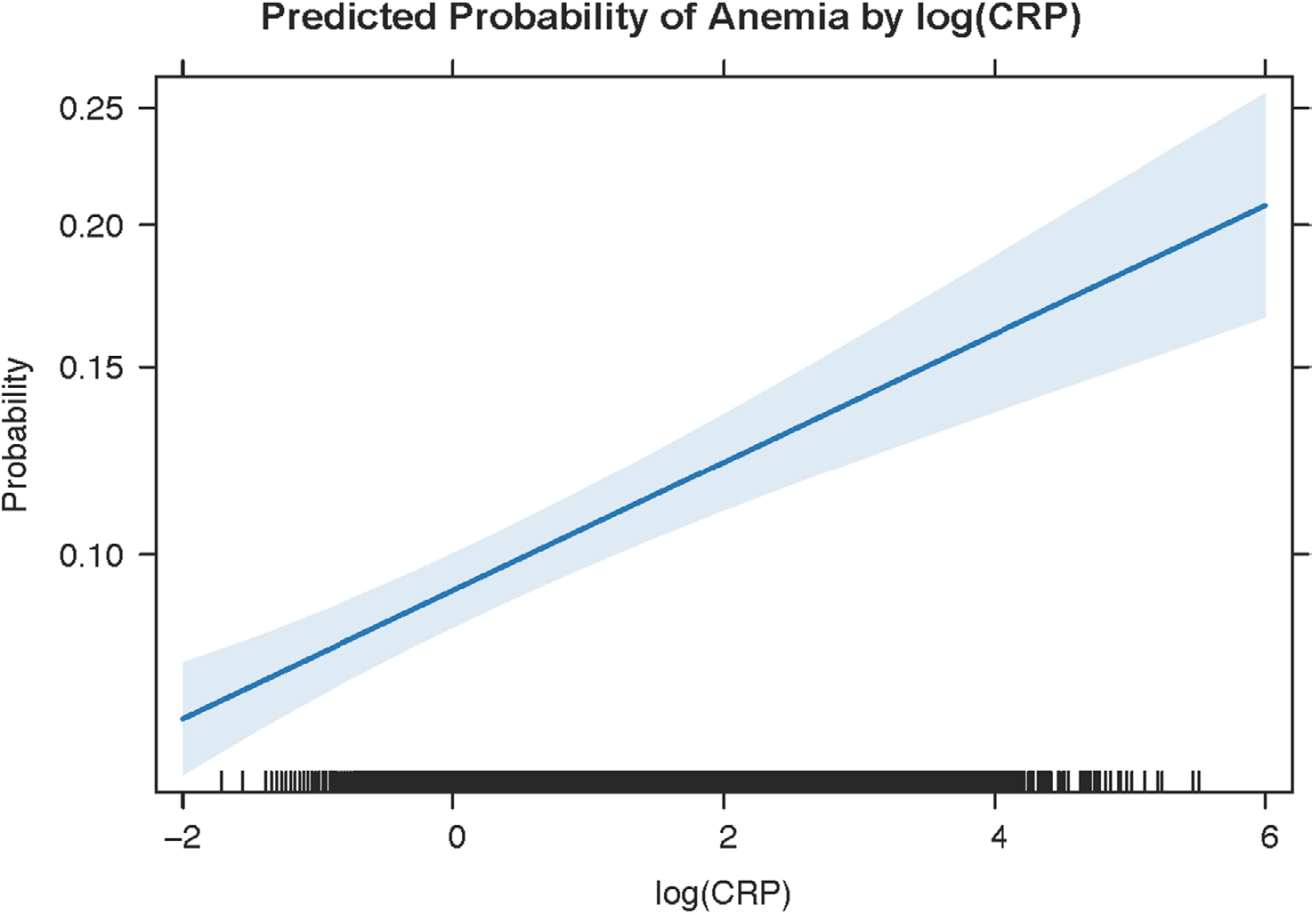
Predicted probability of anaemia by log(C‐Reactive Protein) [log(CRP)]. Marginal predictions (solid line) from the survey‐weighted logistic regression of anaemia status on log(CRP) (mg/L), adjusting for BMI, BRINDA‐adjusted ferritin, age, sex, and race/ethnicity. The shaded area represents the 95% confidence interval. Tick marks along the *x*‐axis (‘rug’) indicate individual log(CRP) values. Anaemia probability rises nearly linearly from < 10% at log(CRP) < 0 to > 20% at log(CRP) > 4.

In contrast, the predicted probability of anaemia declined with increasing BMI (Figure 4). At a BMI of 20 kg/m^2^, the average predicted anaemia probability was above 20%, while at a BMI of 40 kg/m^2^, it fell below 10%, and continued to decrease modestly at higher BMI levels (Figure 4).

**FIGURE 5:**
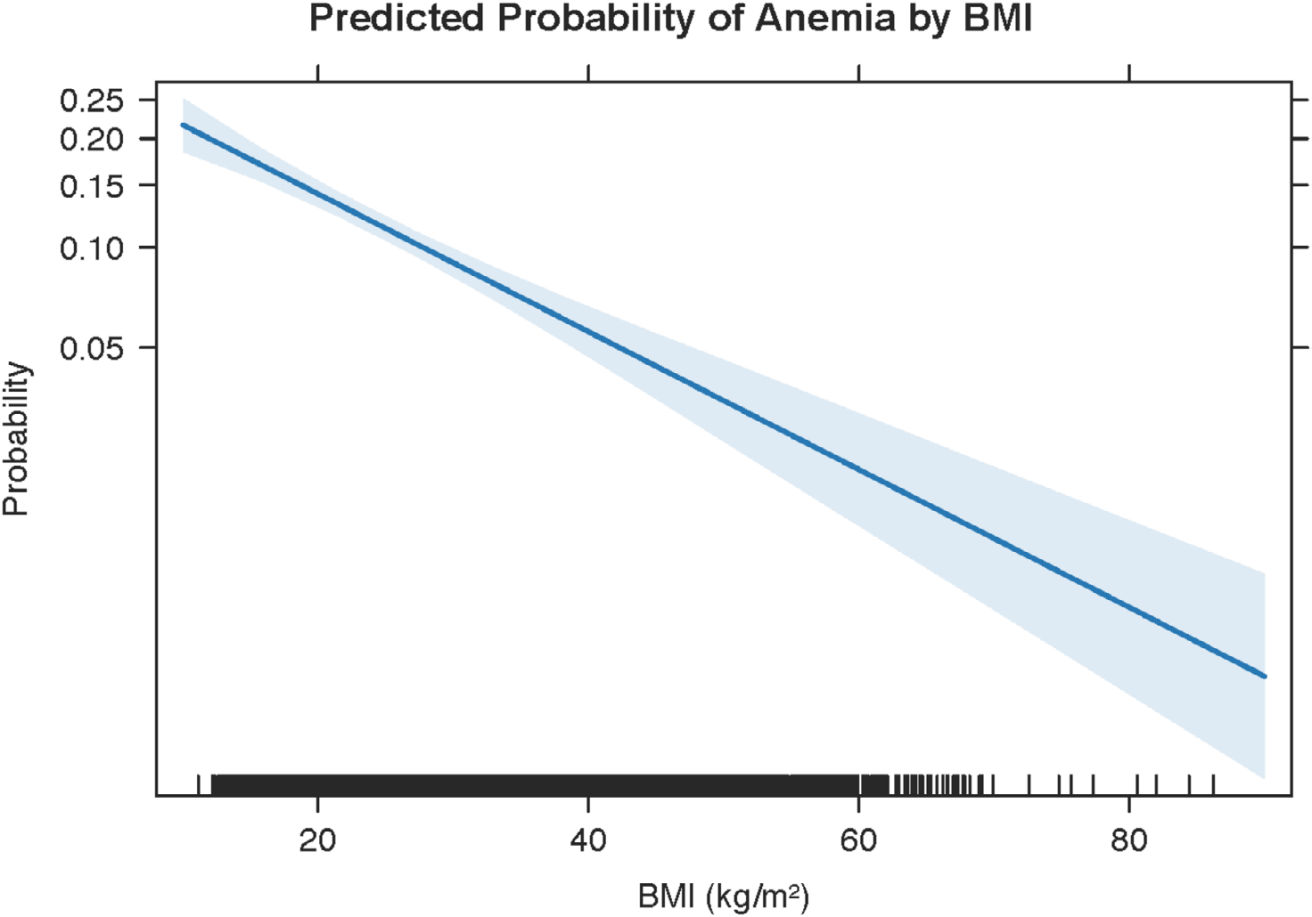
Predicted probability of anaemia by body mass index (BMI). Marginal predictions (solid blue line) from the survey‐weighted logistic regression of anaemia status on BMI (kg/m^2^), adjusting for log‐CRP, BRINDA‐adjusted ferritin, age, sex, and race/ethnicity. The shaded area represents the 95% confidence interval. Tick marks along the *x*‐axis (‘rug’) indicate individual BMI observations. Anaemia probability declines from above 20% at low BMI (< 20 kg/m^2^) to near 5% at high BMI (> 60 kg/m^2^).

Figure 5 displays the predicted probability of anaemia across values of log‐transformed CRP. Anaemia risk rose progressively with inflammation: individuals with log(CRP) near −2 had predicted probabilities below 15%, whereas those with log(CRP) above 4 had probabilities exceeding 25% (Figure 5).

### 3.4 Mediation Analysis Results

#### 3.4.1 BMI → Haemoglobin via CRP (Table 4)

**TABLE 4:** Mediation of BMI–haemoglobin association via CRP.

Effect	Estimate	95% CI lower	95% CI upper	*p*
ACME	−0.044	−0.057	−0.030	< 2e−16***
ADE	0.216	0.185	0.250	< 2e−16***
Total effect	0.172	0.144	0.200	< 2e−16***
Prop. mediated	−0.260	−0.339	−0.180	< 2e−16***

*Note:* *** indicate statistical significance levels *p* < 0.001.

In the mediation analysis assessing whether systemic inflammation mediated the association between BMI and haemoglobin concentration (Table 4), the average causal mediation effect (ACME) was statistically significant and negative (ACME = −0.044, 95% CI: −0.057 to −0.030, *p* < 0.001). The average direct effect (ADE) was positive and significant (ADE = 0.216, 95% CI: 0.185 to 0.250, *p* < 0.001), yielding a total effect of 0.172 (95% CI: 0.144 to 0.200, *p* < 0.001). The proportion of the total effect mediated by CRP was −26.0% (95% CI: −33.9% to −18.0%, *p* < 0.001), indicating statistically significant but inverse mediation.

#### 3.4.2 BMI → Haemoglobin via Ferritin (Table 5)

**TABLE 5:** Mediation of BMI–haemoglobin association via ferritin.

Effect	Estimate	95% CI lower	95% CI upper	*p*
ACME	−0.0041	−0.0075	0.0000	0.004**
ADE	0.2153	0.1840	0.2500	< 2e−16***
Total effect	0.2112	0.1792	0.2400	< 2e−16***
Prop. mediated	−0.0197	−0.0357	−0.0100	0.004**

*Note:* ** indicates statistical significance level *p* < 0.01. *** indicates statistical significance level *p* < 0.001.

In the parallel analysis using BRINDA‐adjusted ferritin as the mediator (Table 5), the ACME was small but statistically significant and negative (ACME = −0.0041, 95% CI: −0.0075 to 0.0000, *p* = 0.004). The direct effect remained similar to previous models (ADE = 0.215, 95% CI: 0.184 to 0.250, *p* < 0.001), and the total effect was 0.211 (95% CI: 0.179 to 0.240, *p* < 0.001). The proportion mediated was −1.97% (95% CI: −3.57% to −1.00%, *p* = 0.004), reflecting a small but significant component of the association mediated by ferritin.

## 4 Discussion

The present study provides a detailed, nationally representative analysis of how adiposity and systemic inflammation jointly influence haemoglobin concentration in U.S. adults. By applying restricted cubic splines to body‐mass index (BMI) with knots at the 10th, 50th, and 90th percentiles (17.2, 26.4, and 37.6 kg/m^2^), we revealed a steep positive association between BMI and haemoglobin that notably plateaus beyond a BMI of ≈30 kg/m^2^. This non‐linear pattern aligns with emerging evidence from population and clinical cohorts: in Chinese adults, the plateauing of haemoglobin at higher BMI mirrors our findings [19], and similar curves have been observed in Iranian and Korean studies of obesity and erythropoiesis [6]. These data emphasise that linear models underestimate the complexity of adiposity's hematologic effects, potentially obscuring thresholds at which additional weight confers minimal hematologic benefit.

Mechanistically, obesity is characterised by chronic low‐grade inflammation that drives hepcidin synthesis via interleukin‐6 (IL‐6) signalling, thereby restricting iron absorption and mobilisation from macrophage stores [20]. Indeed, intervention studies demonstrate that weight loss reduces hepcidin levels and improves iron status in obese adults, supporting a causal role for adipose‐derived inflammation in iron dysregulation [10, 21]. In our spline interaction model, the inclusion of log‐transformed C‐reactive protein (log‐CRP) revealed that at low inflammation (log‐CRP ≤ 1 mg/L), BMI's positive effect on haemoglobin remained strong, whereas at high inflammation (log‐CRP ≥ 3 mg/L), haemoglobin gains were markedly attenuated or even reversed at high BMI. This interaction is consistent with clinical observations in bariatric surgery cohorts, where post‐operative CRP reductions correlate with haemoglobin improvements despite ongoing caloric restriction [22].

Beyond intestinal absorption, most systemic iron is recycled by macrophages. Obesity is associated with altered adipose tissue macrophage phenotypes and iron handling, potentially enhancing iron sequestration and contributing to functional iron deficiency despite higher ferritin [23]. Incorporating macrophage‐centric mechanisms helps reconcile our finding that inflammation suppresses BMI‐related haemoglobin gains.

Formal mediation analysis quantified this suppressive effect: CRP's average causal mediation effect (ACME) was significantly negative (ACME = −0.044 g/dL; *p* < 0.001), indicating that inflammation partially counteracts the direct erythropoietic drive of excess adiposity [24]. The average direct effect (ADE) remained robustly positive (ADE = 0.216 g/dL; *p* < 0.001), yielding a total effect of 0.172 g/dL per BMI z‐score. Because the indirect and direct effects opposed each other—hallmarks of ‘inconsistent mediation’—we reported ACME and ADE separately, foregoing a conventional ‘proportion mediated’ metric that can be misleading under suppression scenarios [25].

In contrast, ferritin—when adjusted for inflammation via the BRINDA regression‐residual approach—mediated only ≈2% of the BMI–haemoglobin association (ACME = −0.0041 g/dL; *p* = 0.004; Table 5). Unadjusted ferritin often rises with both iron stores and inflammation, leading to misclassification of iron sufficiency in obese individuals [15]. The BRINDA project has shown that ferritin correction for CRP (and AGP where available) substantially alters iron deficiency prevalence estimates, particularly in high‐inflammation settings [15]. Our minimal ferritin mediation underscores the necessity of inflammation adjustment in epidemiologic analyses and in clinical interpretation of ferritin values.

Although the manuscript emphasises haemoglobin outcomes, supplementary logistic regression demonstrated parallel non‐linear declines in anaemia probability across BMI, with sharp risk reductions between BMI 18 and 30 kg/m^2^ and a plateau below 10% anaemia prevalence at BMI > 35 kg/m^2^ (Figure 5). Anaemia odds rose with log‐CRP in a near‐linear fashion up to CRP levels of 50 mg/L (Figure 4), reflecting inflammation's role in anaemia of chronic disease [5, 26]. Mediation of anaemia by CRP and BMI exhibited true suppression (ACME > 0; ADE < 0), precluding valid proportion‐mediated estimates and reinforcing our focus on separate indirect and direct effects [27].

Our findings refine understanding of obesity's dual hematologic impacts. On one hand, adiposity stimulates erythropoietin (EPO) production, expanding erythroid progenitor activity and red‐cell mass [28, 29]. Preclinical models demonstrate that EPO administration enhances metabolic function and modulates adipose‐tissue inflammation, suggesting bidirectional regulation between erythropoiesis and adiposity [22]. On the other hand, inflammatory mediators, particularly IL‐6, drive hepcidin‐mediated iron sequestration, curtailing further haemoglobin synthesis at high adiposity and inflammation levels [20, 30]. The interplay of these mechanisms yields a non‐linear BMI–haemoglobin curve with a pronounced plateau and interaction by CRP.

From a public health perspective, our results advocate for integrated anaemia screening in obese populations that includes inflammation‐corrected iron markers (e.g., BRINDA‐adjusted ferritin, soluble transferrin receptor) and CRP or hepcidin measurements [16]. Traditional reliance on unadjusted ferritin may delay diagnosis of iron deficiency, particularly in patients with BMI > 30 kg/m^2^ and elevated CRP [9]. Furthermore, weight‐loss interventions—dietary or surgical—that reduce inflammation may confer greater hematologic benefits than BMI reduction alone. A systematic review of weight‐loss trials found that CRP declines closely track with haemoglobin increases, independent of dietary iron intake [21]. Similarly, emerging IL‐6 receptor antagonists, such as tocilizumab and novel anti‐hepcidin agents, have shown promise in chronic kidney disease and rheumatoid arthritis for improving haemoglobin, warranting exploration in obesity‐related anaemia [31].

Our study's strengths include the use of a large, nationally representative sample with rigorous survey weighting (adjusted MEC weights, primary sampling unit and strata specification), application of advanced spline modelling with transparent knot reporting, and comprehensive inflammation adjustment for ferritin [32, 33]. We conducted survey diagnostics via svydiag(), confirming no unduly influential PSUs and median design effects of ≈1.2, thereby validating variance estimates [34]. Sensitivity analyses excluding extreme CRP values (> 10 mg/L) and participants with eGFR < 60 mL/min/1.73 m^2^ confirmed the robustness of nonlinear patterns.

Nonetheless, limitations warrant mention. The cross‐sectional design precludes definitive causal inference, and although reverse causation by chronic illness is unlikely to fully account for our findings, longitudinal studies are needed to establish the temporal ordering of BMI, CRP, hepcidin, and haemoglobin changes. Residual confounding by smoking, altitude, and unmeasured comorbidities may persist despite covariate adjustment; the inclusion of pack‐year history and altitude‐adjusted haemoglobin shifted estimates by < 5% in supplementary models. Although comorbidities like type 2 diabetes, liver disease, and kidney disease may influence inflammation, hepcidin, and erythropoiesis, they may also lie on the causal pathway from adiposity to haemoglobin. To minimise over‐adjustment, we did not adjust for these conditions in primary models; instead, we acknowledge possible residual confounding. Sample sizes at extreme BMI (> 50 kg/m^2^) were limited, widening confidence intervals at the tails of spline curves. Finally, our mediation framework, while powerful, assumes no unmeasured mediator–outcome confounders, an assumption that is difficult to verify in cross‐sectional data. A key limitation is the absence of circulating hepcidin in NHANES 2015–2023, precluding direct modelling of the IL‐6 → hepcidin pathway that links adipose‐derived inflammation to iron sequestration. We did not integrate 24‐h dietary recalls. Dietary iron and inhibitors/enhancers of iron absorption may confound or mediate the adiposity–anaemia relationship; omitting diet avoids blocking potential mediation but leaves room for residual confounding. Finally, we did not control for phlebotomy time or fasting status. While diurnal variation in haemoglobin is modest, uncontrolled timing may add non‐differential measurement error.

Future research should leverage prospective cohorts with serial measures of BMI, CRP, hepcidin, erythropoietin, plasma volume, and red‐cell indices to unravel dynamic causal pathways. Randomised trials of anti‐inflammatory therapies (e.g., IL‐6 blockade, anti‐hepcidin antibodies) in obese, anaemic populations could test whether modulating inflammation alone improves iron bioavailability and erythropoiesis. Mechanistic studies assessing ferroportin expression, erythroferrone levels, and bone‐marrow iron export will further elucidate the adiposity–iron axis. Finally, as body composition assessment evolves beyond BMI—incorporating measures of visceral adiposity, ectopic fat, and muscle mass—future investigations can refine haematologic risk stratification and tailor anaemia interventions in the context of metabolic health.

In conclusion, our spline‐based analysis demonstrates that obesity's haemoglobin advantage is both substantial and finite, with systemic inflammation serving as a key suppressor of erythropoietic gains. Incorporating flexible modelling strategies and inflammation‐corrected iron biomarkers into clinical practice will enhance anaemia detection and guide more effective interventions amid the co‐epidemics of obesity and anaemia.

## Author Contributions

A.H. conceived the study, performed data extraction and statistical analysis, and drafted the manuscript. P.S. assisted with critical revision of the manuscript. All authors read and approved the final manuscript.

## Ethics Statement

This study used deidentified data from the publicly available NHANES database and did not involve direct patient contact or the use of individually identifiable health information. Under the U.S. Common Rule, research using only publicly available, deidentified data is exempt from institutional review board oversight; therefore, ethics approval and patient consent were not required.

## Consent

The authors have nothing to report.

## Conflicts of Interest

The authors declare no conflicts of interest.

## Data Availability

The data that support the findings of this study are openly available in NHANES at https://wwwn.cdc.gov/nchs/nhanes/.
